# Supplementary material for: ALS/FTD‐associated FUS activates GSK‐3β to disrupt the VAPB–PTPIP51 interaction and ER–mitochondria associations
Source: EMBO Rep. 2016 Jul 14;17(9):1326–42. doi: 10.15252/embr.201541726 (PMC5007559; doi:10.15252/embr.201541726)
Supplement: Supplementary file 1 — Expanded View Figures PDF [file EMBR-17-1326-s001.pdf]

Expanded View Figures

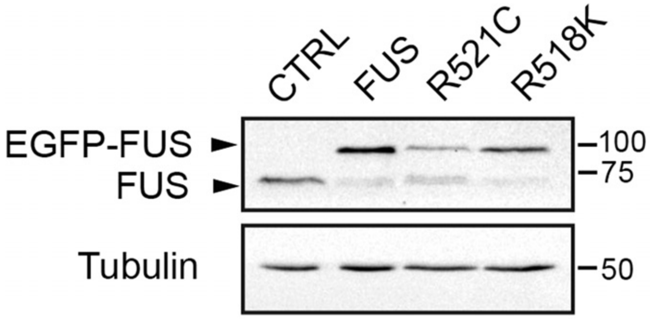

Figure EV1. Expression of EGFP-FUS reduces the expression of endogenous FUS.
HEK293 cells were transfected with control EGFP, EGFP-FUS, EGFP-FUSR521C or EGFP-FUSR518K and 72 h post-transfection, the samples were probed on immunoblots for FUS (using FUS antibody) and tubulin as a loading control.

 © 2016 The Authors

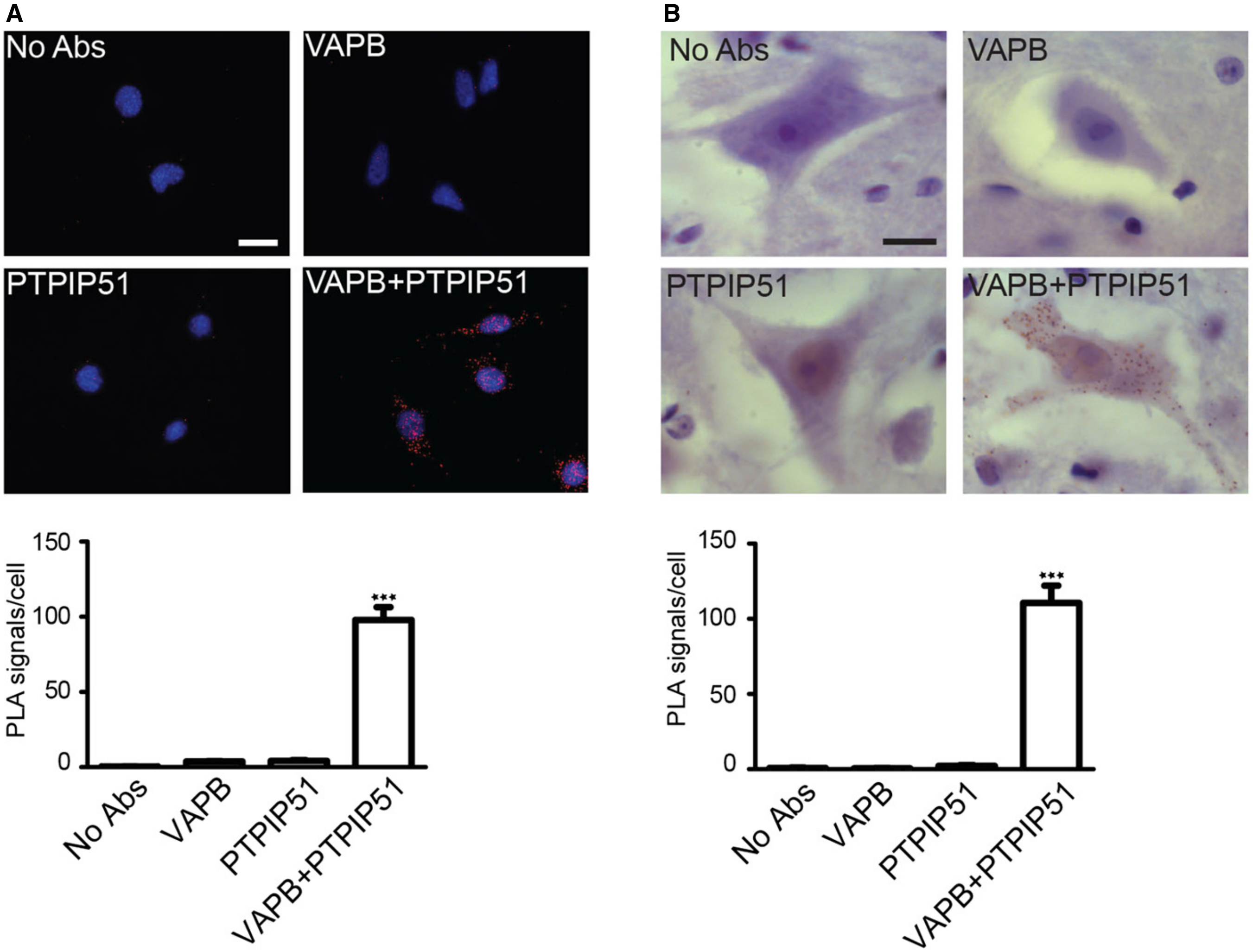

Figure EV2. Control experiments involving omission of primary antibodies demonstrate the specificity of the VAPB-PTPIP51 proximity ligation assays.

A, B Panel (A) shows NSC34 cells; panel (B) shows mice spinal cords. Samples were probed with no primary antibodies (no Abs), VAPB only, PTPIP51 only or VAPB + PTPIP51 antibodies. In (A) samples are counterstained with DAPI to show nuclei. Scale bar = 10 μm (A) and 30 μm (B). Bar charts show proximity signals/cell. Data were analysed by one-way ANOVA and Tukey's *post hoc* test. $N = 16$ cells (A) and 12 cells (B), error bars are s.e.m.; ***$P < 0.001$.

© 2016 The Authors

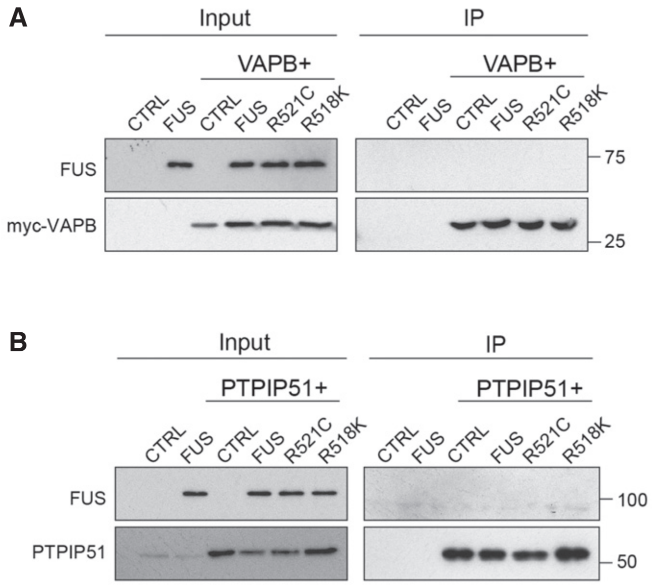

Figure EV3. FUS does not bind VAPB or PTPIP51 in immunoprecipitation assays from transfected HEK293 cells.

A Cells were transfected as indicated with control vector (CTRL), HA-FUS + CTRL, myc-VAPB + CTRL, or myc-VAPB + either HA-FUS, HA-FUSR521C or HA-FUSR518K. VAPB was immunoprecipitated via the myc-tag and the samples probed on immunoblots for VAPB using rabbit VAPB antibody and for co-immunoprecipitating FUS via the HA tag. Input VAPB and FUS were detected using myc and HA antibodies.

B Cells were transfected as indicated with control vector (CTRL), HA-FUS + CTRL, HA-PTPIP51 + CTRL or HA-PTPIP51 + either HA-FUS, HA-FUSR521C or HA-FUSR518K. PTPIP51 was immunoprecipitated using rat anti-PTPIP51 and the samples probed for PTPIP51 using rabbit anti-HA antibody and for co-immunoprecipitating FUS using rabbit FUS antibody. Input PTPIP51 and FUS were detected using PTPIP51 and EGFP antibodies, respectively.

 © 2016 The Authors
